# Increased Expression of *KNSTRN* in Lung Adenocarcinoma Predicts Poor Prognosis: A Bioinformatics Analysis Based on TCGA Data

**DOI:** 10.7150/jca.51591

**Published:** 2021-04-02

**Authors:** Pengbo Deng, Rongrong Zhou, Jinghui Zhang, Liming Cao

**Affiliations:** 1 Department of Respiratory Medicine, National Key Clinical Specialty, Branch of National Clinical Research Center for Respiratory Disease, Xiangya Hospital, Central South University, Changsha, China.; 2 Hunan Provincial Clinical Research Center for Respiratory Diseases, Changsha, China.; 3 Xiangya Hospital, Central South University, Changsha, China.; 4 National Clinical Research Center for Geriatric Disorders, Xiangya Hospital, Central South University, Changsha, China.; 5 Department of Radiation Oncology, Xiangya Hospital, Central South University, Changsha, China.

**Keywords:** Bioinformatics analysis, lung adenocarcinoma, kinetochore-localized astrin/SPAG5-binding protein (*KNSTRN*), prognosis, the Cancer Genome Atlas (TCGA).

## Abstract

**Purpose:** Available evidence indicates that kinetochore-localized astrin/SPAG5-binding protein (*KNSTRN*) is an oncogene in skin carcinoma. This study aimed to evaluate the prognostic value of *KNSTRN* in lung adenocarcinoma (LUAD) underlying the Cancer Genome Atlas (TCGA) database.

**Methods:** The relationship between clinicopathological features and *KNSTRN* was analyzed with the Wilcoxon signed-rank test and logistic regression. The clinicopathological characteristics associated with overall survival (OS) were evaluated using Cox regression and the Kaplan-Meier method. Gene ontology (GO) analysis, gene set enrichment analysis (GSEA), and single-sample GSEA (ssGSEA) were performed using TCGA data.

**Results:** The *KNSTRN* expression level was found to be significantly higher in LUAD tissue than in normal lung tissue. Also, it correlated significantly with advanced clinicopathological characteristics. The Kaplan-Meier survival curve revealed a significant relationship of high expression of *KNSTRN* with poor OS in patients with LUAD. The multivariate Cox regression hazard model demonstrated the *KNSTRN* expression level as an independent prognostic factor for patients with LUAD. GO and GSEA analyses indicated the involvement of *KNSTRN* in cell cycle checkpoints, DNA replication, and G2-M checkpoint M phase. Based on ssGSEA analysis, *KNSTRN* had a positive relationship with Th2 cells and CD56^dim^ natural killer cells. The *KNSTRN* expression levels in several types of immune cells were significantly different.

**Conclusion:** The findings suggested that the increased expression level of *KNSTRN* was significantly associated with the progression of LUAD and could also serve as a novel prognostic biomarker for patients with LUAD.

## Introduction

Lung cancer is still the leading cause of cancer-related death in both men and women worldwide 1. Lung adenocarcinoma (LUAD) is the most common subtype of lung cancer leading to more than 1 million deaths annually across the world 2. Although the treatment of LUAD has significantly improved over the past decades with the development and progress of new therapeutic methods, the 5-year survival rate for patients with LUAD remains very low, approximately 5% 3. This may be attributed to the difficulty in the early diagnosis and the lack of appropriate therapeutic approaches for LUAD. Thus, finding effective biomarkers of LUAD for early diagnosis and targeted therapy is crucial to increase the survival rate.

The kinetochore-localized astrin/SPAG5-binding protein (*KNSTRN*) gene, also known as a small kinetochore-associated protein gene 4, which encodes for a kinetochore-associated protein, is found to promote the metaphase-to-anaphase transition and chromosome segregation during mitosis 4. It is also believed to be closely related to tumorigenesis in the skin. A genomic study by Lee et al. identified recurrent somatic mutations of *KNSTRN* in 19% of cutaneous squamous cell carcinoma (SCC) 5. Among the detected mutations of *KNSTRN*, more than half of them mapped to a 17-amino-acid N-terminal region, with a “hotspot” serine-to-phenylalanine substitution present at codon 24 (p.Ser24Phe). In addition, *KNSTRN* mutations were found in 23 of 490 (4.7%) melanomas, and the p.Ser24Phe mutation was found in 19% actinic keratosis (AKs) 5. Thus, a mutated *KNSTRN* gene, especially the p.Ser24Phe mutation, might functionally lead to disrupted chromatid cohesion in normal cells and correlated with increased aneuploidy in primary tumors and enhanced SCC development *in vivo* 5. Additionally, Schmitz et al. recently reported that another recurrent somatic mutation “p.Ala40Glu” in the *KNSTRN* gene was associated with basal proliferative AK lesions and invasive carcinoma 6. Moreover, in a recent study on endometrial cancer, it was found that the expression of KNSTRN was positively correlated with AKT1, and high expression of KNSTRN was significantly associated with poor prognosis of endometrial cancer. And as is well-known, the PI3K/AKT/mTOR pathway can promote the proliferation of endometrial cancer cells 7. Therefore, based on the above findings, it is possible to speculate that genetic alternations of *KNSTRN*, the essential molecular for cell-cycle progression, may be related to the proliferation of cancer cells. Recent studies also found that altered *KNSTRN* expression resulted in the loss of chromatid cohesion in the noncutaneous tumor cell line HeLa cells. These data suggested a possibility that aberrant *KNSTRN* expression might exist in other tissues as well as in the skin, and played an important role in tumorigenesis.

Researches have indicated that sustaining cell proliferation is one of the basic traits of lung cancer 8, 9. Dysregulation of signaling pathways involved in cell proliferation, including PI3K/AKT/mTOR, have also been observed in lung cancer 9. And in our pre-analysis using the RNA sequencing data of LUAD patients in TCGA database, we found several differentially expressed genes including *KNSTRN*. However, the role of *KNSTRN* in LUAD tumorigenesis and the potential molecules or pathways involved need to been clarified.

Thus, the objective of the present study was to evaluate the prognostic value of *KNSTRN* expression in human LUAD based on data obtained from the Cancer Genome Atlas (TCGA) database. To gain further insight into the potential functions, the biological pathways involved in LUAD pathogenesis-related *KNSTRN* regulatory network, Gene Ontology (GO) analysis and gene set enrichment analysis (GSEA) were performed. Moreover, the relationship of *KNSTRN* with tumor-infiltrating immune cells in different tumor microenvironments was analyzed using single-sample gene set enrichment analysis (ssGSEA).

## Methods

### RNA-sequencing data acquisition and processing

Clinical information of lung adenocarcinoma patients and high-throughput RNA-sequencing data were downloaded from the TCGA database (https://portal.gdc.cancer.gov). There was a total of 522 cases of lung adenocarcinoma. Eventually, 513 patients were enrolled into analysis due to eligibility of both clinical information as well as RNA-seq data. The transcript expression levels were estimated using the fragments per kilobase per million fragments mapped (FPKM) method in HTSeq. Also, the RNA-Seq gene expression level 3 HTSeq-FPKM data of 513 patients with LUAD and clinical data were converted into transcripts per million (TPM) reads format for further analysis.

### Differential expression analysis

According to the median value normalized by the *Z*-score, tumors were divided into high- and low-*KNSTRN* expression groups, and differentially expressed genes were analyzed by HTSeq-Counts using the DESeq2 package 10. The log fold change (logFC) > 2 and the adjusted *P* value < 0.01 were set as the thresholds for a statistical difference. The differential analysis results were displayed using volcano plots and heat maps.

### Enrichment analysis

The Metascape (http://metascape.org) 11 database was used for the GO enrichment analysis of *KNSTRN* and its list of differentially expressed molecules, including biological processes, molecular functions, and cellular components. The parameters were set at *P* < 0.01, minimum count > 3, and enrichment factor > 1.5. The GSEA 12 method enriched *KNSTRN* expression-related pathways and ranked the genome 1000 times per analysis. The C2.all.v6.2.symbols.gmt composition was used as the reference gene set. The threshold for statistically significant GSEA analysis was set to a corrected *P* < 0.05 and an FDR < 0.25. The results of the enrichment analysis were characterized using corrected *P* values and normalized enrichment scores (NESs). The Cluster Profiler package 13 was used for GSEA enrichment analysis and visualization.

### Immune infiltration analysis

The marker genes of 24 different immune cell types were acquired from Bindea G's research 14. The infiltration of 24 immune cell types in the tumor was analyzed using the ssGSEA method. The Spearman correlation method was used to analyze the degree of correlation between *KNSTRN* and the aforementioned 24 types of immune cells, and for the analysis of immune cell infiltration between *KNSTRN* high- and low-expression groups.

### Statistical analysis

All statistical analyses were performed in R (v3.6.2). The Wilcoxon rank-sum test was used for unpaired samples, while the Wilcoxon signed-rank test was used for paired samples. The receiver operating characteristic (ROC) curve was used to analyze whether *KNSTRN* expression could be the diagnostic marker. Kruskal-Wallis test, Wilcoxon signed-rank test, and logistic regression method were used to analyze the relationship between clinicopathological characteristics and *KNSTRN* expression. The chi-square test or Fisher exact test was used to analyze the relationship between KNSTRN expression and clinicopathological characteristics. Cox hazard regression analysis or Kaplan-Meier method was used to evaluate the prognostic value of *KNSTRN* expression. In the Cox hazard regression analysis, variables with *P* < 0.1 in the univariate analysis were included in the multivariate Cox hazard regression. A statistically significant *P* value was set at 0.05. Due to the incomplete clinical information in the TCGA database, not every sample recorded clinical baseline information such as age, TNM stage, treatment outcome, and etc. Therefore, a complete analysis of each clinical category was not possible. So, there is a discrepancy between the total number of samples and the number of samples in different clinical categories in the tables of Results section.

## Results

### Relationship between *KNSTRN* expression and clinical characteristics

**Figure 1:**
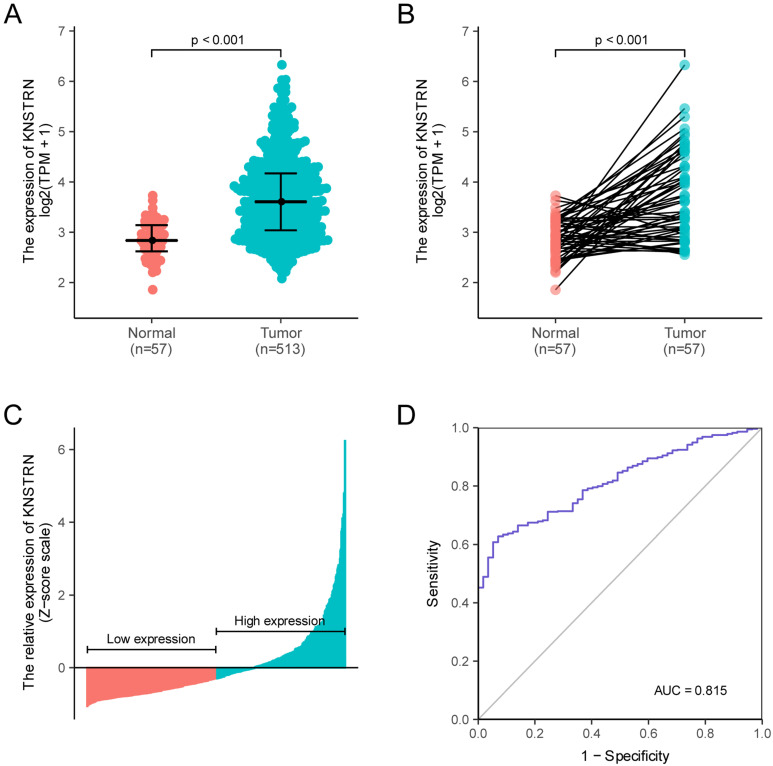
** Relationship between *KNSTRN* expression and lung adenocarcinoma (LUAD).** (A) Differential expression analysis: Analysis of differences in the *KNSTRN* expression levels between tumor tissues and normal tissues. (B) Analysis of differential expression of *KNSTRN* between tumor tissues and matched paracancerous tissues. (C) *KNSTRN* expression status in all LUAD samples between low-expression and high-expression groups. (D) Diagnostic value of *KNSTRN* expression in LUAD. Analysis between two groups of unpaired samples: Wilcoxon rank-sum test; analysis between two groups of paired samples: Wilcoxon signed-rank test.

*KNSTRN* expression in LUAD and normal tissues was analyzed, revealing a difference in the *KNSTRN* expression levels in LUAD and normal tissues (Supplementary Fig. 1A, B and C); *KNSTRN* was highly expressed in tumor tissues (*P* < 0.001, Fig. 1A). At the same time, *KNSTRN* expression in LUAD tissues and paired adjacent nontumorous tissues were analyzed. The results also suggested that *KNSTRN* was highly expressed in tumor tissues (*P* < 0.001, Fig. 1B). *KNSTRN* expression in tumor tissues was standardized using the *Z*-score, and the LUAD cohort was divided into high- and low-expression groups according to *KNSTRN* expression (Fig. 1C). In addition, ROC curves were used to analyze the diagnostic value of *KNSTRN*. The area under the curve (AUC) of *KNSTRN* was 0.815, and the results suggested that *KNSTRN* might be a potential diagnostic biomarker (Fig. 1D).

**Figure 2:**
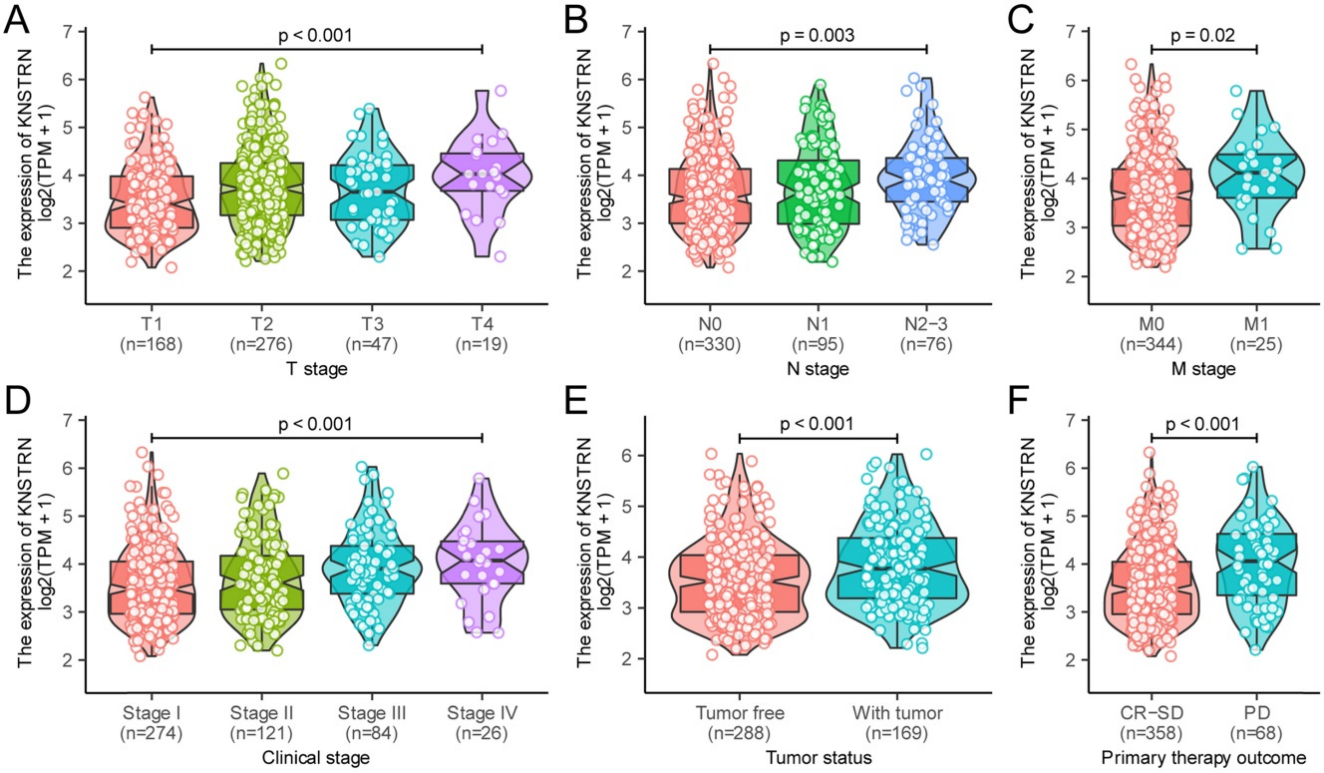
** Relationship between *KNSTRN* expression and clinicopathological characteristics.** Relationship of *KNSTRN* expression with T stage (A), N stage (B), M stage (C), clinical stage (D), tumor status (E), and primary therapy outcome (F). Analysis between two groups: Wilcoxon rank-sum test; analysis between multiple groups: Kruskal-Wallis rank-sum test.

In addition, the Kruskal-Wallis test and Wilcoxon signed-rank test were used to analyze the relationship between *KNSTRN* expression and clinical characteristics. The increased expression levels of* KNSTRN* positively correlated with higher grades of T stage (*P* < 0.001, Fig. 2A), N stage (*P* = 0.003, Fig. 2B), M stage (*P* = 0.02, Fig. 2C), clinical stage (*P* < 0.001, Fig. 2D), tumor status (*P* < 0.001, Fig. 2E), and the outcome of the primary therapy (*P* = 0.003, Fig. 2F). At the same time, consistent results were also found using the chi-square test or Fisher exact test (Table 1). Moreover, the univariate logistic regression of *KNSTRN* expression also suggested a close relationship between *KNSTRN* and clinical characteristics., including T stage [odds ratio (OR) = 1.04 (1.02-1.07), *P* < 0.001], N stage [OR = 1.02 (1.01-1.04), *P* = 0.010], clinical stage [OR = 1.03 (1.01-1.05), *P* = 0.003)], tumor status [OR = 1.03 (1.01-1.05), *P* = 0.002], outcome of the primary therapy [OR = 1.04 (1.02-1.07), *P* < 0.001], and *TP53* mutation [OR = 1.04 (1.02-1.06), *P* < 0.001] (Table 2). No significant difference was found in the relationship with M stage [OR = 1.02 (0.99-1.05), *P* = 0.099]. These results suggested that *KNSTRN* expression was related to clinical characteristics.

### Prognostic value of *KNSTRN* expression in LUAD

**Figure 3:**
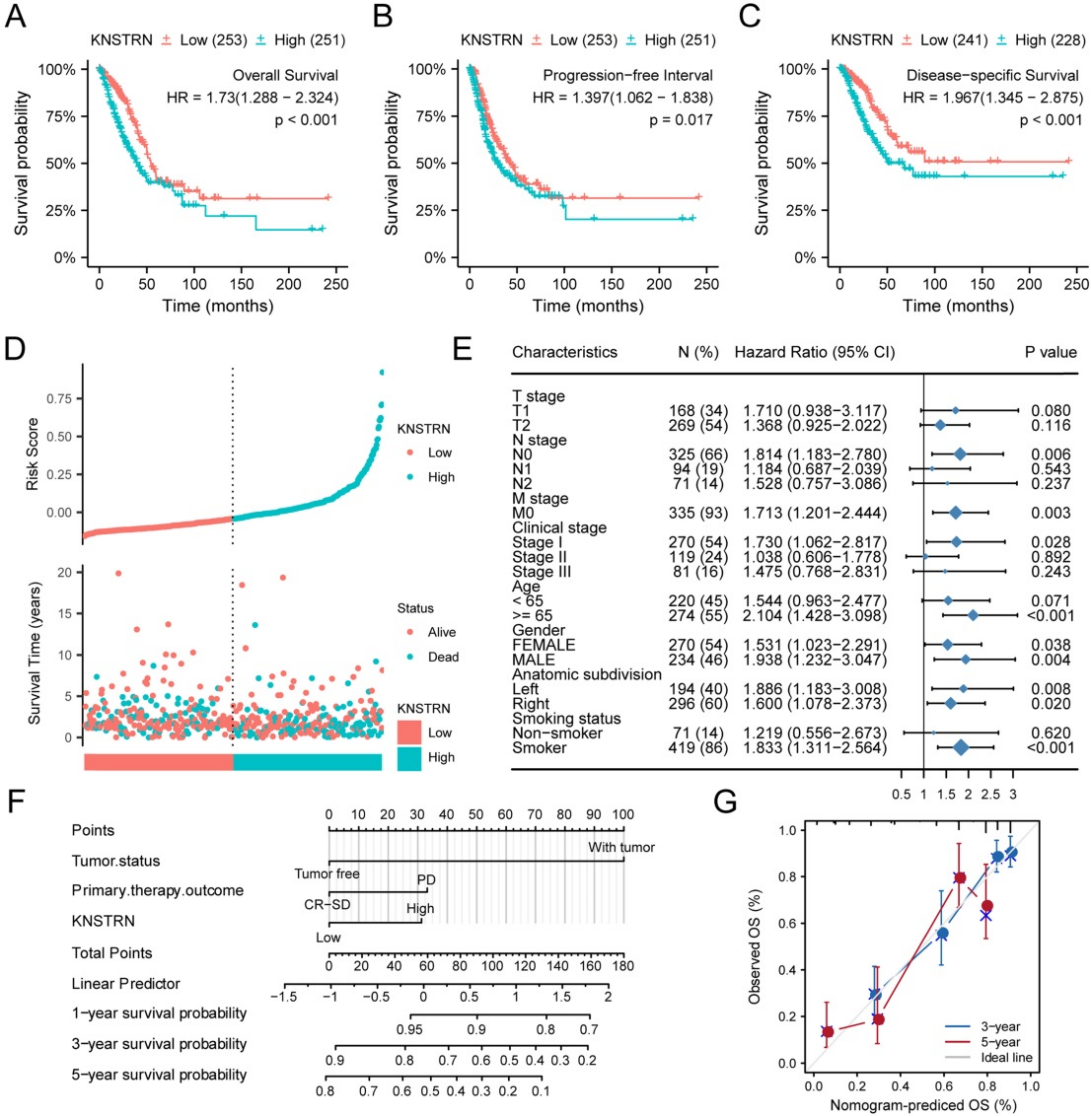
** Prognostic analysis of *KSTRN* expression.** Patients with high expression level of *KNSTRN* had poor prognosis compared with those with low expression level of *KNSTRN*, including overall Survival (OS) (A), progression-free interval (PFS) (B), and disease-specific survival (DSS) (C) (both log-rank *P* < 0.001). (D) Risk factors for OS of *KNSTRN* expression. (E) Prognosis of *KNSTRN* expression in subgroups of clinical characteristics (OS). (F) Multivariate analysis nomogram based on the clinical characteristics of *KNSTRN* expression. (G) Calibration chart shows the predictive performance of the model constructed using multifactor Cox regression analysis.

The relationships of *KNSTRN* expression with prognostic outcomes in overall survival (OS), progression-free survival (PFS), and disease-specific survival (DSS) are shown in Figure 3A-3C. High expression of *KNSTRN* was associated with poor OS [hazard ratio (HR) = 1.730 (1.288-2.324), *P* < 0.001, Fig. 3A (and Supplementary Fig. 1D, E and F)], poor PFS [HR = 1.397 (1.062-1.838), *P* = 0.017, Fig. 3B], and poor DSS [HR = 1.967 (1.345-2.875), *P* < 0.001, Fig. 3C]. At the same time, this study also showed the distribution of high expression and low expression of *KNST*R*N* with respect to OS and risk score (Fig. 3D). The results suggested that patients with LUAD and high-risk scores had high expression levels of *KNSTRN*, while patients with low-risk scores were associated with low expression levels of *KNSTRN*.

In addition, this study analyzed the relationship between *KNSTRN* expression and different subgroups. *KNSTRN* was found to be highly expressed in N0 stage [HR = 1.814 (1.183-2.780), *P* = 0.006], M0 stage [HR = 1.713 (1.201-2.444), *P* = 0.003], stage I [HR = 1.730 (1.062-2.817), *P* = 0.028], age >65 years [HR = 2.104 (1.428-3.098), *P* < 0.001], and smokers [HR = 1.833 (1.311-2.564), *P* < 0.001]; high expression of* KNSTRN* was related to poor OS (Table 3 and Fig. 3E).

Moreover, univariate Cox regression was also performed, and the results suggested that TNM stage, clinical stage, tumor status, primary therapy outcome, and high expression of *KNSTRN* were associated with poor OS (*P* < 0.05). Moreover, high expression of *KNSTRN* was an independent prognostic factor for OS [HR = 1.730 (1.288-2.234), *P* < 0.001, Table 4] as revealed by multivariate Cox hazard regression analysis. Tumor status, primary therapy outcome, and *KNSTRN* expression were used to construct a clinical prognostic risk score for LUAD (Fig. 3F). At the same time, the prediction accuracy of the model was assessed using a calibration chart (Fig. 3G). The results suggested that the *KNSTRN* expression level could better predict the 3-year and 5-year survival of patients. In general, all these results suggested that the *KNSTRN* expression level correlated with the prognosis of patients with LUAD.

### Relationship between *KNSTRN* expression and whole gene expression profile

**Figure 4:**
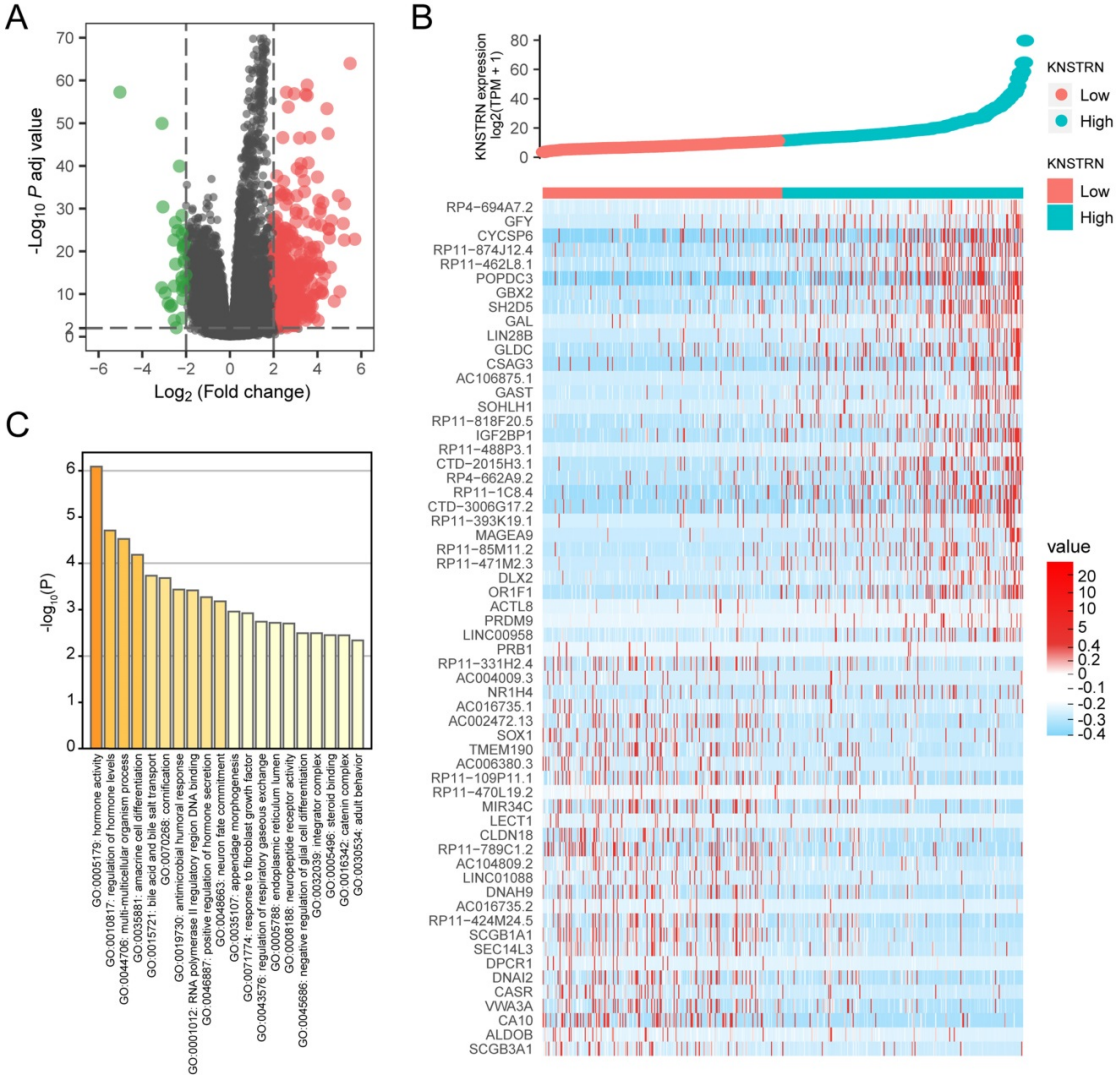
** Differential expression analysis and GO enrichment analysis related to *KSTRN* gene expression.** (A) Volcanic plot of differentially expressed based on *KSTRN* expression status. (B) Heat map showing 30 upregulated and downregulated genes, selected based on *KSTRN* expression status. (C) Metascape database was used to analyze the GO enrichment results of differentially expressed genes screened based on *KSTRN* expression.

The gene expression profiling analysis related to *KNSTRN* was performed to further explore the biological function of *KNSTRN* in LUAD. A total of 34 downregulated genes and 486 upregulated genes were considered to be significantly associated with *KNSTRN* expression (logFC > 2 and *P*adj < 0.01) (Fig. 4A). Further, the top 30 upregulated genes and top 30 downregulated genes among these abnormally expressed genes were shown in the gene expression heat map (Fig. 4B). In addition, based on *KNSTRN* expression, GO enrichment analysis was performed using Metascape. The biological functions of the *KNSTRN* gene are associated mainly with hormone activity, regulation of hormone levels, multi-multicellular organism process, and so forth (Fig. 4C).

### GSEA analysis of *KNSTRN* expression

**Figure 5:**
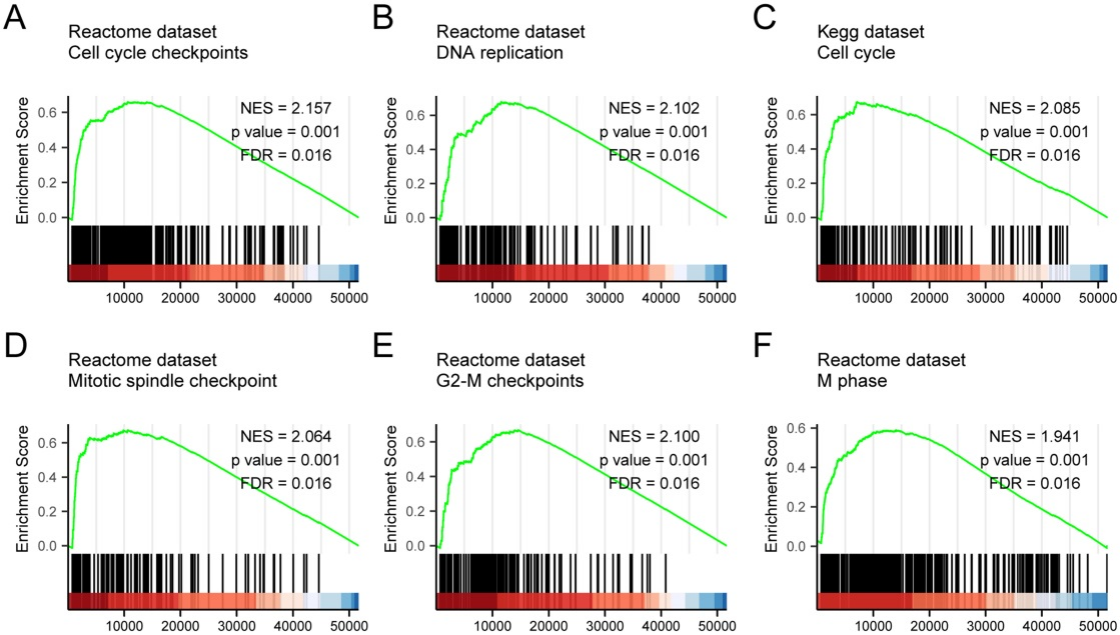
** GSEA enrichment analysis results.** GSEA results showed that cell cycle checkpoints (A), DNA replication (B), cell cycle (C), mitotic spindle checkpoint (D), G2-M checkpoint 9 (E), and M phase (F) were enriched mainly in *KSTRN*-related LUAD. ES, Enrichment score; FDR, false discovery rate; NES, normalized ES.

GSEA analysis of the TCGA gene expression data was used to identify functional and biological pathways between low and high expression of *KNSTRN*. Based on the normalized enrichment scores (NESs), the enrichment signaling pathway most significant in terms of *KNSTRN* gene expression was selected (Fig. 5 and Table 5). GSEA analysis results showed that the highly expressed *KNSTRN* phenotype was concentrated mainly in cell cycle checkpoints (A), DNA replication (B), cell cycle (C), mitotic spindle checkpoint (D), G2-M checkpoint 9 (E), and M phase (F).

### Relationship between *KNSTRN* expression and immune infiltration

**Figure 6:**
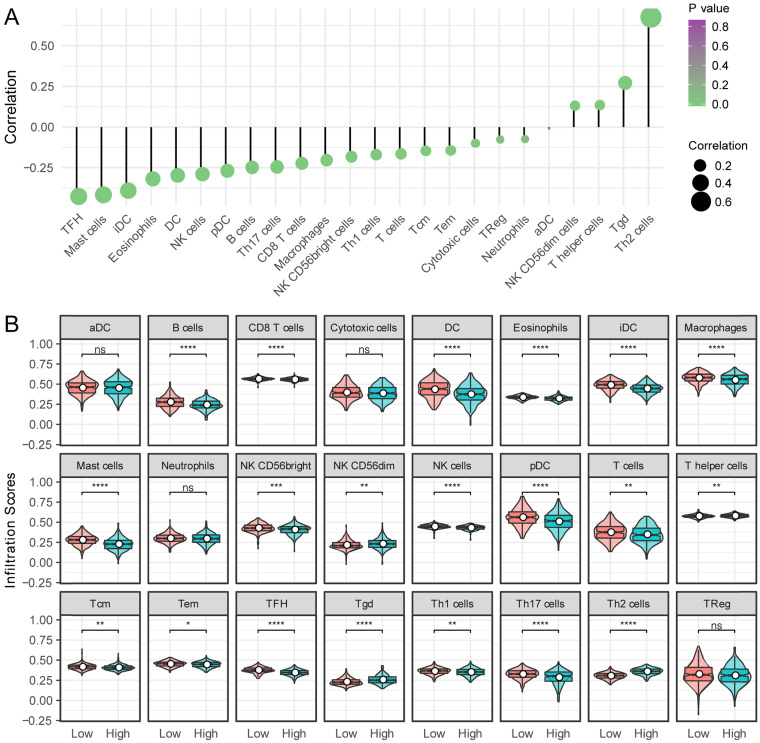
** Relationship between *KSTRN* expression and immune infiltration.** (A) Relationship between *KSTRN* expression and immune cells. (B) Enrichment differences in different immune cell subpopulations in the high- and low-expression groups of *KSTRN*. Nonsignificant (ns) represents *P* ≥ 0.05; ^*^represents *P* <0.05; ^**^represents *P* <0.01; ^***^represents *P* <0.001; ^****^represents *P* <0.0001.

Next, the relationship between *KNSTRN* expression and 24 different immune cell types was evaluated in LUAD. *KNSTRN* expression had a close positive relationship with T helper 2 (Th2) cells, Tgd, and NK CD56dim cells, and a close negative relationship with T follicular helper (TFH) cells, mast cells, immature dendritic cells (iDCs), and so forth (Fig. 6A). Further research showed significant differences in the *KNSTRN* expression level among the infiltrating immune cells, including B cells, CD8 T cells, eosinophils, macrophages, TFH, TH2 cells, NK cells, and so on (Fig. 6B).

## Discussion

LUAD is a highly malignant and heterogeneous disease with varied prognosis. Despite extensive studies on the biomarkers for lung cancer, research on the prognostic markers of LUAD were still limited 15. Therefore, novel biomarkers with clinicopathological significance and prognostic value for LUAD needed to be urgently identified. In this study, bioinformatics analysis was conducted based on the data obtained from the TCGA database to study the significance, prognostic value, and hypothetical mechanism of *KNSTRN* in LUAD.

Previous studies showed that KNSTRN was a mitosis-associated protein that contributed to chromosome alignment, accurate chromosome segregation, and maintenance of spindle pole architecture 16-18. The overexpression of KNSTRN could promote cell apoptosis after exposure to tumor necrosis factor alpha (TNF-α), TNF-related apoptosis-inducing ligand (TRAIL), staurosporine, and ultraviolet irradiation 19. Furthermore, mutant *KNSTRN* was reported to be involved in the pathogenesis of basal cell carcinoma 20, endometrial cancer 21, and mucosal melanoma 22. In addition, Lee et al. performed whole-exome sequencing on cutaneous SCC samples and patient-matched normal skin samples to investigate the genetic causes of cutaneous SCC 5. The results showed that* KNSTRN* ranked third after *CDKN2A* and *TP53* among frequently mutated genes in SCC, providing evidence that *KNSTRN* was a novel oncogene not reported earlier. Moreover, they searched TCGA database, suggesting that *KNSTRN* might also play a role in melanoma. However, the expression and role of *KNSTRN* in lung cancer were not reported. In the present study, bioinformatics analysis using high-throughput RNA-sequencing data from TCGA database demonstrated that *KNSTRN* was significantly highly expressed in LUAD tissues compared with paired normal tissues, indicating that* KNSTRN* played a role in tumorigenesis and progression. In addition, the ROC analysis showed that the AUC was 0.815 in the diagnosis of LUAD, suggesting that *KNSTRN* might be a potential diagnostic biomarker. Furthermore, high expression of *KNSTRN* positively correlated with advanced clinicopathological characteristics (TNM stage, clinical stage, tumor status, and primary therapy outcome), survival time, and poor prognosis. Additionally, the subgroup analysis indicated that *KNSTRN* expression could be used to stratify the prognosis of patients with stage I LUAD. It might also be useful when identifying stage I patients with undesirable prognoses and subsequently guiding their therapeutic regimen. *KNSTRN* was further validated as an independent prognostic factor for OS in multivariate analysis. In summary, *KNSTRN* overexpression was associated with cancer progression and poor prognosis in LUAD. It also found to be a diagnostic marker and might be used in the early screening of LUAD.

The genomic analysis of 2229 patients within 18 different tumor types, including lung squamous cell carcinomas, could not detect any somatic mutation of *KNSTRN* (p.Ser24Phe) 23. However, other variations of *KNSTRN*, such as overexpression, might be involved in the development of cancer. With respect to the molecular mechanism involved, the STRING website (https://string-db.org/) was employed to predict the potential genes' interaction with *KNSTRN*. The corresponding protein-protein interaction (PPI) network of KNSTRN was constructed when we selected the interactions pertaining to Homo sapiens, chose physical network and showed minimum interactions with a medium confidence = 0.4 (Supplementary Fig. 1G). It was found that CENPE, CENPL, PRPF19, and KIF2B had the tendency to interact with KNSTRN, which were all involved in the mitosis. Interestingly, Huo et al. performed a multi-steps bioinformatics analysis in endometrial cancer, and the identified six hub genes, including *KNSTRN*, were in the PPI network with AKT1, and both higher expression of AKT1 and KNSTRN was significantly associated with poor prognosis of endometrial cancer 21. In addition, GO enrichment analysis using Metascape in this study found that the biological functions of *KNSTRN* were associated mainly with hormone activity, regulation of hormone levels, and multi-multicellular organism process. To further investigate the functions of *KNSTRN* in LUAD, GSEA using TCGA data. The findings showed that cell cycle checkpoints, DNA replication, cell cycle, mitotic spindle checkpoint, G2-M checkpoint, and M phase are differentially enriched in the *KNSTRN* high-expression phenotype. These results were highly consistent with previous findings. However, the role of *KNSTRN* in these pathways needs to be further verified in LUAD, both *in vitro* and *in vivo*.

Another important aspect of this study was that *KNSTRN* expression correlated with diverse immune infiltration levels in LUAD. Numerous studies have demonstrated that the lung tumor microenvironment contributes to the immunological changes during the progression of lung cancer 24, and there are various known and unknown mechanisms involved in this process. Sharfe et al. recently reported that the dual loss of p110δ PI3-kinase and KNSTRN expression led to combined immunodeficiency and multisystem syndromic features 25. This finding suggested that KNSTRN might play an important role in the immune system. Thus, the difference in immune cell infiltration between patients with high and low *KNSTRN* expression was compared in this study. The results based on ssGSEA analysis demonstrated a significantly positive relationship of the *KNSTRN* expression level with the infiltration level of Th2 cells, Tgd, and NK CD56dim cells, and a significantly negative relationship of the infiltration level of TFH cells, mast cells, and iDC with *KNSTRN* expression. Moreover, these relationships indicated the role of *KNSTRN* in regulating tumor immunology in LUAD. In addition, the results showed significant differences in the *KNSTRN* expression level in the immune cells, including B cells, CD8 T cells, T helper cells (Th1, Th2, TFH, and Th17), memory T cells (Tc and Tem), NK cells, DC (iDC and pDC), eosinophils, macrophages, neutrophils, and mast cells. Together these findings suggested that *KNSTRN* might play an important role in regulating immune functions in LUAD.

Since KNSTRN was recognized as an oncogene only recently, the present study was performed to investigate its relationship with different progression steps and gradings of LUAD. However, a limitation of this study was that only one dataset was included, and it was not validated in our own clinical samples. Thus, further experimental verifications are necessary to elucidate the biological functions of these predicted molecular mechanisms in LUAD to deepen our understanding of the direct impact of KNSTRN on LUAD. Moreover, the prognostic value of *KNSTRN* in LUAD also needs further verification.

In summary, the data showed aberrantly increased expression levels of *KNSTRN* in LUAD tissues compared with normal lung tissues. High expression of *KNSTRN* in patients with LUAD was significantly associated with advanced clinicopathological characteristics. Survival analyses indicated that high expression of *KNSTRN* could serve as an independent factor for poor survival in patients with LUAD. Furthermore, the *KNSTRN* high-expression phenotype was associated with cell cycle checkpoints, DNA replication, cell cycle, mitotic spindle checkpoint, G2-M checkpoint, and M phase, as revealed by GSEA putatively via physical interactions of CENPE, CENPL, PRPF19, and KIF2B. Taken together, the findings suggested that *KNSTRN* could be a novel prognostic biomarker for patients with LUAD. However, the mechanisms by which *KNSTRN* promotes tumor progression and metastases in LUAD need further elucidation.

## Supplementary Material

Supplementary figure S1.

## Figures and Tables

**Table 1:** Relationship between *KNSTRN* expression and clinicopathological characteristics in patients with lung adenocarcinoma.

Characters	level	Low expression of *KNSTRN*	High expression of *KNSTRN*	*p*	test
n		256	257		
OS event (%)	Alive	180 (70.3)	146 (56.8)	0.002	
	Dead	76 (29.7)	111 (43.2)		
T stage (%)	T1	104 (40.6)	64 (24.9)	<0.001	exact
	T2	125 (48.8)	151 (58.8)		
	T3	21 (8.2)	26 (10.1)		
	T4	4 (1.6)	15 (5.8)		
	NA	2 (0.8)	1 (0.4)		
N stage (%)	N0	179 (69.9)	151 (58.8)	0.002	exact
	N1	45 (17.6)	50 (19.4)		
	N2	23 (9.0)	51 (19.8)		
	N3	1 (0.4)	1 (0.4)		
	NA	8 (3.1)	4 (1.6)		
M stage (%)	M0	164 (64.1)	180 (70.0)	0.090	
	M1	7 (2.7)	18 (7.0)		
	NA	85 (33.2)	59 (23.0)		
Clinical stage (%)	Stage I	156 (60.9)	118 (45.9)	<0.001	
	Stage II	60 (23.4)	61 (23.7)		
	Stage III	27 (10.6)	57 (22.2)		
	Stage IV	8 (3.1)	18 (7.0)		
	NA	5 (2.0)	3 (1.2)		
Tumor status (%)	Tumor free	157 (61.3)	131 (51.0)	0.080	
	With tumor	77 (30.1)	92 (35.8)		
	NA	22 (8.6)	34 (13.2)		
Primary therapy outcome (%)	PD	23 (9.0)	45 (17.5)	0.003	exact
	SD	23 (9.0)	14 (5.4)		
	PR	4 (1.6)	2 (0.8)		
	CR	177 (69.1)	138 (53.7)		
	NA	29 (11.3)	58 (22.6)		
Age (%)	< 65	98 (38.3)	122 (47.4)	0.025	
	>= 65	151 (59.0)	123 (47.9)		
	NA	7 (2.7)	12 (4.7)		
Gender (%)	Female	158 (61.7)	118 (45.9)	<0.001	
	Male	98 (38.3)	139 (54.1)		
Anatomic subdivision (%)	Left	107 (41.8)	92 (35.8)	0.257	
	Right	144 (56.2)	155 (60.3)		
	NA	5 (2.0)	10 (3.9)		
Smoking status (%)	Non-smoker	46 (18.0)	28 (10.9)	0.028	
	Smoker	202 (78.9)	223 (86.8)		
	NA	8 (3.1)	6 (2.3)		
TP53 mutation (%)	No	165 (64.4)	102 (39.7)	<0.001	
	Yes	89 (34.8)	152 (59.1)		
	NA	2 (0.8)	3 (1.2)		

OS: Overall Survival; NA: Not Available

**Table 2:** Logistic regression analysis of *KNSTRN* expression.

Characteristics	Total (N)	Odds ratio in *KNSTRN* expression	*P* value
T stage (T1 vs. T2-4)	510	1.04 (1.02-1.07)	**<0.001**
N stage (N0 vs. N1-3)	501	1.02 (1.01-1.04)	**0.01**
M stage (M0 vs. M1)	369	1.02 (0.99-1.05)	0.099
Clinical stage (Stage I vs. Stage II-IV)	505	1.03 (1.01-1.05)	**0.003**
Tumor status (Tumor free vs. With tumor)	457	1.03 (1.01-1.05)	**0.002**
Primary therapy outcome (CR-SD vs. PD)	426	1.04 (1.02-1.07)	**<0.001**
Anatomic subdivision (Left vs. Right)	498	1.01 (0.99-1.02)	0.547
TP53 mutation (No vs. Yes)	508	1.04 (1.02-1.06)	**<0.001**

**Table 3:** Prognostic analysis of *KNSTRN* expression in a subset of patients with LUAD.

Characteristics	N (%)	Hazard ratio	*P* value
T stage			
T1	168 (34)	1.710 (0.938-3.117)	0.080
T2	269 (54)	1.368 (0.925-2.022)	0.116
T3	45 (9)	2.930 (1.166-7.363)	0.022
N stage			
N0	325 (66)	1.814 (1.183-2.780)	0.006
N1	94 (19)	1.184 (0.687-2.039)	0.543
N2	71 (14)	1.528 (0.757-3.086)	0.237
M stage			
M0	335 (93)	1.713 (1.201-2.444)	0.003
Clinical stage			
Stage I	270 (54)	1.730 (1.062-2.817)	0.028
Stage II	119 (24)	1.038 (0.606-1.778)	0.892
Stage III	81 (16)	1.475 (0.768-2.831)	0.243
Age			
< 65	220 (45)	1.544 (0.963-2.477)	0.071
>= 65	274 (55)	2.104 (1.428-3.098)	<0.001
Gender			
Female	270 (54)	1.531 (1.023-2.291)	0.038
Male	234 (46)	1.938 (1.232-3.047)	0.004
Anatomic subdivision			
Left	194 (40)	1.886 (1.183-3.008)	0.008
Right	296 (60)	1.600 (1.078-2.373)	0.020
Smoking status			
Non-smoker	71 (14)	1.219 (0.556-2.673)	0.620
Smoker	419 (86)	1.833 (1.311-2.564)	<0.001

**Table 4:** Univariate and multivariate Cox proportional hazard analyses of *KNSTRN* expression.

Characteristics	Univariate analysis		Multivariate analysis	
	HR (95% CI)	*P* value	HR (95% CI)	*P* value
T stage (T1 vs. T2-4)	1.668 (1.184-2.349)	0.003	1.096 (0.641-1.874)	0.739
N stage (N0 vs. N1-3)	2.606 (1.939-3.503)	<0.001	1.495 (0.739-3.026)	0.263
M stage (M0 vs. M1)	2.111 (1.232-3.616)	0.007	0.92 (0.394-2.15)	0.848
Clinical stage (Stage I vs. Stage II-IV)	2.975 (2.188-4.045)	<0.001	1.033 (0.477-2.24)	0.934
Tumor status (Tumor free vs. With tumor)	6.215 (4.261-9.064)	<0.001	5.483 (3.244-9.266)	<0.001
Primary therapy outcome (CR-SD vs. PD)	3.978 (2.785-5.682)	<0.001	2.478 (1.536-3.997)	<0.001
Age (< 65 vs. >= 65)	1.172 (0.871-1.578)	0.295		
Gender (Female vs. Male)	1.06 (0.792-1.418)	0.694		
Anatomic subdivision (Left vs. Right)	1.024 (0.758-1.383)	0.878		
Smoking status (Non-smoker vs. Smoker)	0.887 (0.587-1.339)	0.568		
KNSTRN (Low vs. High)	1.73 (1.288-2.324)	<0.001	1.563 (1.014-2.409)	0.043

**Table 5:** GSEA enrichment analysis results.

ID	Set Size	Enrichment Score	NES	*P* value	*P* adjust	FDR	Rank	Leading_edge
REACTOME M PHASE	352	0.590	1.941	0.001	0.020	0.016	14014	tags=56%, list=27%, signal=41%
REACTOME CELL CYCLE CHECKPOINTS	263	0.660	2.157	0.001	0.020	0.016	10642	tags=55%, list=21%, signal=44%
REACTOME G2 M CHECKPOINTS	145	0.668	2.100	0.001	0.020	0.016	14627	tags=75%, list=28%, signal=54%
REACTOME DNA REPLICATION	125	0.679	2.102	0.001	0.020	0.016	11562	tags=67%, list=22%, signal=52%
KEGG CELL CYCLE	124	0.675	2.085	0.001	0.020	0.016	7088	tags=44%, list=14%, signal=38%
REACTOME MITOTIC SPINDLE CHECKPOINT	106	0.674	2.064	0.001	0.020	0.016	10642	tags=55%, list=21%, signal=44%
