# Supplementary material for: Increased Expression of KNSTRN in Lung Adenocarcinoma Predicts Poor Prognosis: A Bioinformatics Analysis Based on TCGA Data
Source: J Cancer. 2021 Apr 2;12(11):3239–48. doi: 10.7150/jca.51591 (PMC8100810; doi:10.7150/jca.51591)
Supplement: Supplementary file 1 — Supplementary figure S1. [file jcav12p3239s1.pdf]

Supplementary Figure 1. (A) Mutations of *KNSTRN* gene in 586 patients with lung adenocarcinoma from TCGA (Firehose Legacy) were verified in the cBioPortal database (http://www.cbioportal.org/), and 4% of lung adenocarcinoma patients were found to have KNSTRN mutations. (B) The GEPIA database (http://gepia.cancer-pku.cn/) was used to analyze the distribution of *KNSTRN* in human tissues and the expression changes in tumors. (C) The expression of *KNSTRN* in different cancers was analyzed in GEPIA database. (D) The OS analysis was verified in the GEPIA database. (E) The OS analysis was verified in the KM Plotter database (http://kmplot.com/analysis/). (F) The OS analysis was verified in the GEO database (Data set: GSE72094 [https://www.ncbi.nlm.nih.gov/geo /query/acc.cgi?acc=GSE72094]). (G) Protein-protein interaction plot of the correlation of *KNSTRN* and potentially related genes.

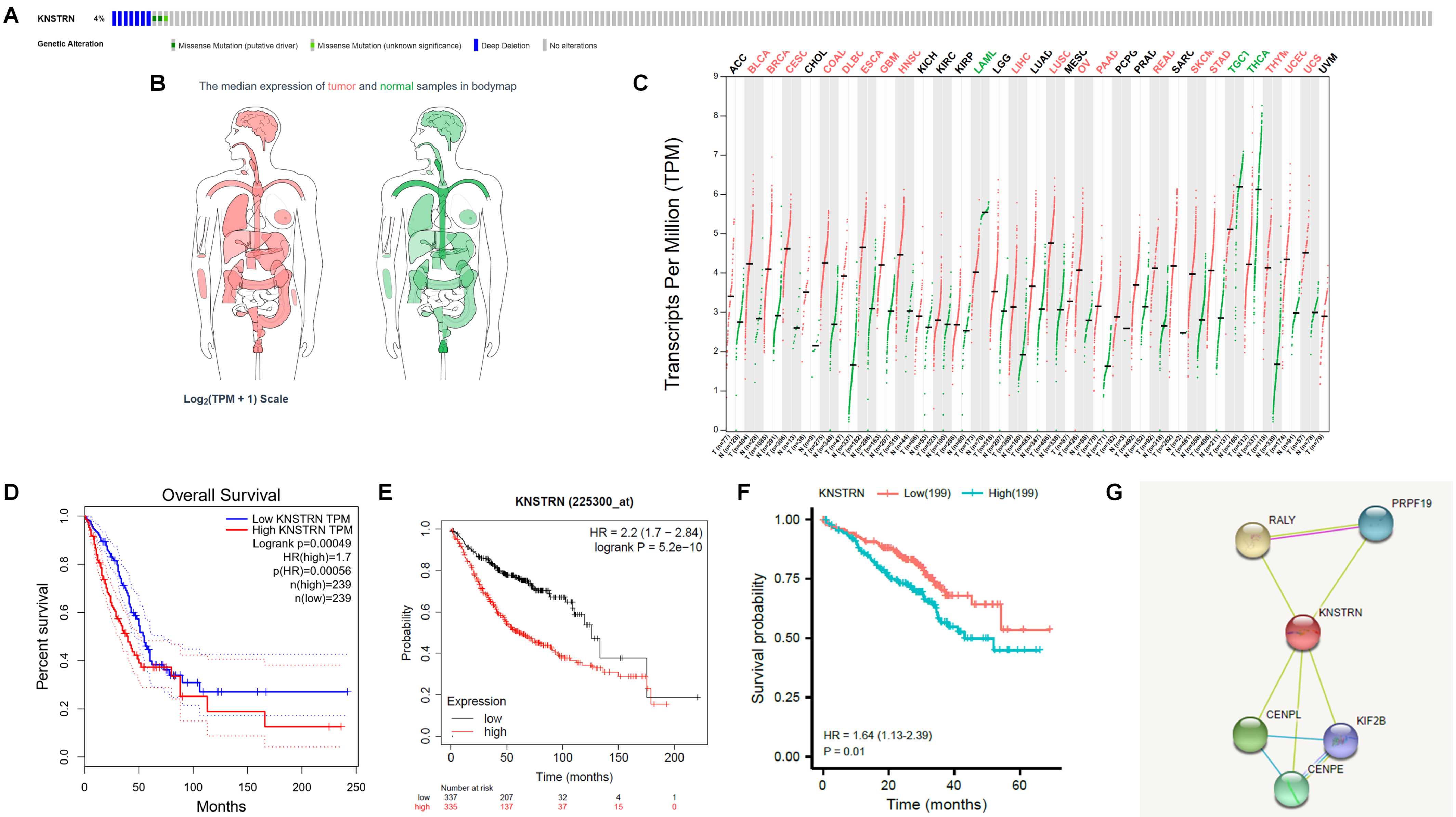
A
KNSTRN 4%
Genetic Alteration
Missense Mutation (putative driver)
Missense Mutation (unknown significance)
Deep Deletion
No alterations
B
The median expression of tumor and normal samples in bodymap
Log2(TPM + 1) Scale
C
Transcripts Per Million (TPM)
ACC BLCA BRCA CESC CHOL COAD DLBC ESCA GBM HNSC KICH KIRC KIRP LAML LGG LIHC LUAD LUSC MESO OV PAAD PCPG PRAD READ SARC SKCM STAD TGCT THCA THYM UCEC UCS UVM
D
Overall Survival
Low KNSTRN TPM
High KNSTRN TPM
Logrank p=0.00049
HR(high)=1.7
p(HR)=0.00056
n(high)=239
n(low)=239
Percent survival
Months
E
KNSTRN (225300_at)
HR = 2.2 (1.7 − 2.84)
logrank P = 5.2e−10
Probability
Expression
low
high
Time (months)
Number at risk
low 337 207 32 4 1
high 335 137 37 15 0
F
KNSTRN Low(199) High(199)
Survival probability
HR = 1.64 (1.13-2.39)
P = 0.01
Time (months)
G
RALY
PRPF19
KNSTRN
CENPL
KIF2B
CENPE
